# Ultrasound-Guided High-Intensity Focused Ultrasound Combined With PD-1 Blockade in Patients With Liver Metastases From Lung Cancer: Protocol for a Single-Arm Phase 2 Trial

**DOI:** 10.2196/59152

**Published:** 2024-11-29

**Authors:** Chao Hu, Qiang Fu, Fei Fei Gao, Jian Zeng, Wei Xiao, Hui Li, Li Peng, Xi Huang, Li Yang, Wen Zhi Chen, Ming Yan Jiang

**Affiliations:** 1 Respiratory Department Xiangtan Central Hospital Xiangtan China; 2 Haifu Micro Noninvasive Center Xiangtan Central Hospital Xiangtan China; 3 State Key Laboratory of Ultrasound in Medicine and Engineering Chongqing Medical University Chongqing China

**Keywords:** high-intensity focused ultrasound, programmed cell death protein, PD-1 blockade, liver metastases, lung cancer, immunotherapy, treatment efficacy, quality of life, HILL study

## Abstract

**Background:**

While immunotherapy has revolutionized oncological management, its efficacy in lung cancer patients with liver metastases remains limited, potentially due to the unique immunosuppressive microenvironment of the liver. Local liver treatment has been shown to enhance the immunotherapy response, and high-intensity focused ultrasound (HIFU), a minimally invasive local treatment, has demonstrated promising results in combination with immunotherapy. However, clinical data regarding HIFU in lung cancer with liver metastases are limited.

**Objective:**

We designed the HILL (Ultrasound-Guided High-Intensity Focused Ultrasound Combined With PD-1 Blockade in Patients With Liver Metastases From Lung Cancer) study to investigate the effectiveness and safety of HIFU in combination with immunotherapy for lung cancer with liver metastases.

**Methods:**

The HILL study is a single-armed, single-center, phase 2 clinical trial that will enroll 30 patients with lung cancer and liver metastases. The treatment regimen involves administering HIFU to liver metastases 1 week before the first dose of a programmed cell death protein (PD)–1 blockade, which is then administered every 3 weeks. The primary aim is to determine the overall response rate based on immune-related response criteria. Secondary aims include safety, progression-free survival, overall response, overall survival, and quality of life. Exploratory studies will also be conducted using whole blood, plasma, archival cancer tissue, and tumor biopsies during progression or relapse to identify potential biomarkers.

**Results:**

The study was funded on March 14, 2022, and received ethical approval on April 27, 2022. Clinical trial registration was completed by June 10, 2022, with participant recruitment beginning on July 10, 2022. Data collection commenced on July 14, 2022, with the enrollment of the first patient. By April 2024, 6 participants had been recruited. The results are expected to be published in December 2026.

**Conclusions:**

This study seeks to improve treatment outcomes for lung cancer patients with liver metastases by combining HIFU and PD-1 inhibition. The study also aims to identify potential biomarkers through exploratory research that can aid in selecting patients for optimized outcomes in the future.

**Trial Registration:**

Chinese Clinical Trial Registry ChiCTR2200061076; https://www.chictr.org.cn/showproj.html?proj=170967

**International Registered Report Identifier (IRRID):**

DERR1-10.2196/59152

## Introduction

Lung cancer is the second most frequently diagnosed tumor and the largest cause of cancer-related fatalities globally, accounting for roughly 1 in 10 (11.4%, 2.2 million) new cancer cases and 1 in 5 (18%, 1.8 million) deaths annually [1,2]. Immunotherapy, a landmark breakthrough in cancer treatment, has revolutionized the field of oncology, contributing to a recent accelerated decline in lung cancer mortality [3,4]. Encouraging clinical trial results with immune checkpoint blockade (ICB) have demonstrated the efficacy of this approach in lung cancer, leading the US Food and Drug Administration to approve programmed cell death protein (PD)–1 inhibitors for all stage III to stage IV lung cancers [3].

Nevertheless, a substantial proportion of patients fail to benefit from immunotherapy, particularly those with lung cancer liver metastases [5]. The existence of baseline liver metastases in patients with lung cancer has been connected with a reduced response to immunotherapy. In the Checkmate 017 and Checkmate 057 trials, the 3-year overall survival (OS) rate for subgroups of patients with lung cancer liver metastases treated with pembrolizumab was 8%, as compared to a 3-year OS of 17% across all enrolled patients [6]. Similarly, in the Keynote 001 trial among patients with non–small cell lung cancer (NSCLC), liver metastasis was linked with a decreased treatment response and shorter progression free survival (PFS) in comparison to patients without liver metastasis [7]. In patients with NSCLC with similar PD-L1 expression, tumor burden, and mutational burden, the presence of liver metastases was linked with reduced OS regardless of age, sex, or treatment modality. [8,9]. Liver metastases are independently associated with a poorer response to immunotherapy, primarily due to the liver’s unique immunosuppressive microenvironment [10]. The liver processes a vast array of harmless dietary antigens daily, necessitating a state of immune tolerance [11]. Metastatic lesions in the liver further enhance this immunosuppressive milieu. Liver metastases highly express integrin receptors, including cluster of differentiation [CD] 44 and lymphocyte function–associated antigen-1, which promotes the sequestration of activated peripheral T cells into the liver, thereby reducing T cell distribution abnormalities [12]. Additionally, liver-infiltrating myeloid cells in metastatic lesions induce T cell apoptosis via the Fas-FasL pathway, resulting in T cell depletion [13]. This extensive antigen-specific T cell exhaustion drives immunosuppression in preclinical models and cancer patients with liver metastases, leading to the enlargement of metastatic lesions and a poor response to immunotherapy [14].

Local treatment of liver metastases may be practical to counter this tumor-permissive immune microenvironment. Radiation therapy (RT) that targets the liver can both inhibit immune-suppressing myeloid cells and boost T cell immunity [13,15]. A preclinical study showed that mice treated with a combination of RT and immunotherapy demonstrated remission of both liver and subcutaneous tumors and considerably increased survival [13]. In another report, a White patient aged 64 years with chemotherapy-resistant metastatic lung adenocarcinoma who received local RT to one of the liver metastases combined with immunotherapy generated a therapeutically effective antitumor immune response [16]. Therefore, we propose that localized therapies targeting hepatic metastases, such as RT, may enhance the efficacy of ICB in patients with liver metastases.

Based on an understanding of tumor–immune system interactions, oncologists categorize tumors as “hot” (high immune cell infiltration, high immune scoring) or “cold” (low infiltration, low immune scoring) based on the type, density, and location of immune cell infiltration. This classification has been validated in patients with tumor, demonstrating its predictive value for tumor recurrence and survival outcomes [17]. In “cold” tumors, the efficacy of ICB is markedly poor, often resulting in no response or excessive adverse reactions [18]. High-intensity focused ultrasound (HIFU) can create a favorable temporal window for immunotherapy to turn previously immunologically “cold” tumors into immune-reactive “hot” tumors. HIFU uses specialized ultrasonic focusing equipment to generate focused ultrasound beams that penetrate the skin and tissue to form focal points deep within the body. At these focal points, the ultrasound intensity is extremely high, generating heat that can rapidly elevate the temperature of exposed tissues above 60°C. When this exposure lasts longer than 1 second, most cells within the tissue undergo immediate irreversible death, minimizing potential damage to surrounding nontarget tissues [19]. The thermal ablation effect of HIFU induces coagulative necrosis in tumor tissues by moving the focal point, thereby rapidly reducing tumor burden and locally releasing tumor antigens. Unlike RT, HIFU can be safely reapplied without increasing systemic toxicity risks, allowing clinicians to administer multiple local treatments even in the event of local tumor recurrence.

**Figure 1:**
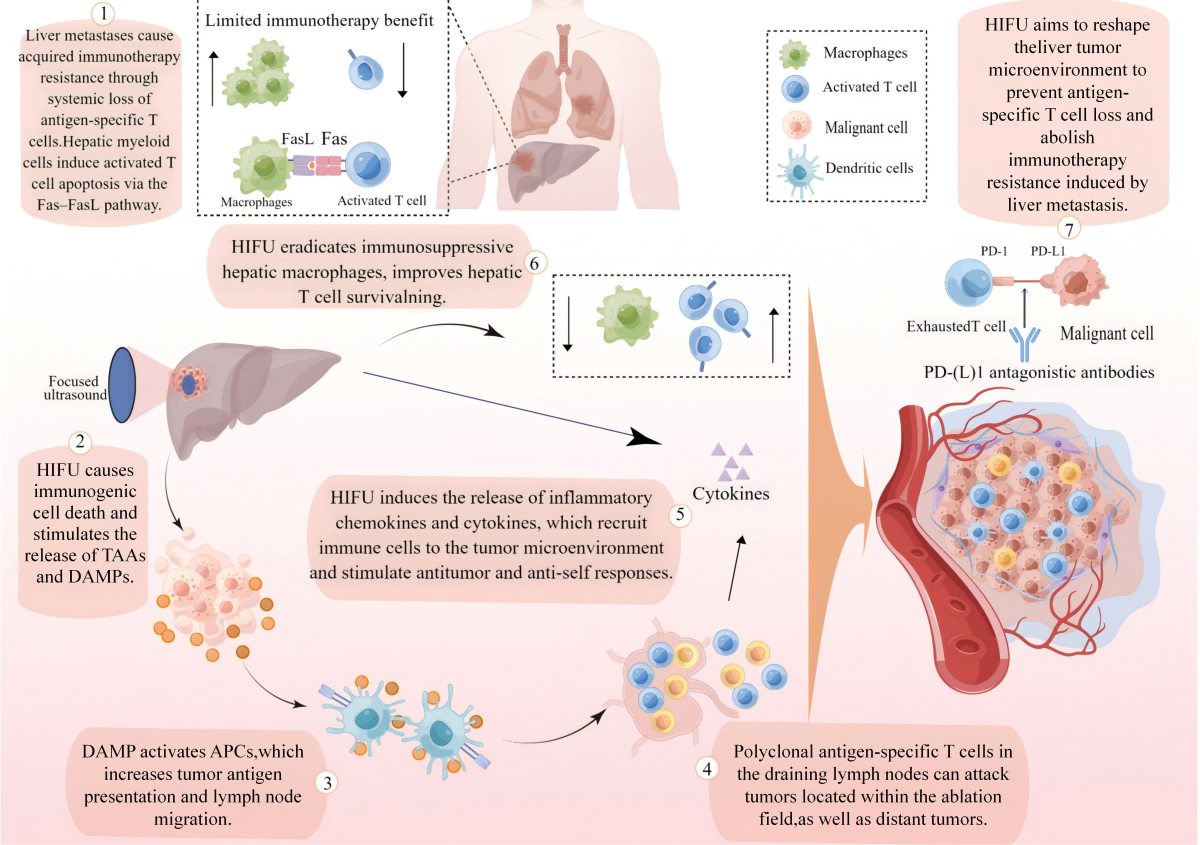
Hypothesis diagram of the research mechanism of HIFU enhancing immunity. HIFU, high-intensive focus ultrasound; TAAs, Tumor Associated Antigens; DAMPs, damage associated molecular patterns; APC, Antigen Presenting Cells.

As a method for real-time imaging and noninvasive local thermal ablation, HIFU is being developed as an alternative to conventional oncologic therapy for a variety of solid malignant tumors, such as liver, kidney, and bone tumors [20-22]. In a rat tumor model of refractory neuroblastoma, HIFU in combination with immunotherapy significantly improved the antitumor response, increasing the survival rate from 0% to 62.5% [23]. The combination’s proposed mechanism of action is shown in Figure 1. In a syngeneic mouse model of epithelial breast cancer, Silvestrini et al [24] also found that immunotherapy combined with HIFU treatment could significantly reduce the number of macrophages and bone- and marrow-derived suppressor cells in tumor tissues, and it could promote the polarization of monocytes to the M1 type. In addition, it significantly upregulated PD-L1 in CD45+ cells and CD8+ T cells generated by IFN-γ, thereby enhancing antitumor immunity.

However, data on the combination of HIFU and immunotherapy in patients with lung cancer with liver metastases is limited. The HILL (Ultrasound-Guided High-Intensity Focused Ultrasound Combined With PD-1 Blockade in Patients With Liver Metastases From Lung Cancer) study intends to investigate the therapeutic efficacy and safety of PD-1 blockade in conjunction with HIFU.

## Methods

### Ethical Considerations

The trial has received ethics approval from Xiangtan Central Hospital Ethics Committee (2022-04-001). All participants provide full informed consent to participate in HILL. The data of the enrolled participants were kept strictly confidential and were anonymized for deidentification. The participants are exempted from any fees for ultrasonic monitoring. The trial is conducted in conformity with the Declaration of Helsinki and Good Clinical Practice guidelines.

### Study Design

The HILL study is a single-center, single-arm, phase 2 trial conducted with lung cancer patients with liver metastases (Chinese Clinical Trial Registry ChiCTR2200061076). Recruitment for the research began on July 10, 2022, and it is expected to be completed in June 2025.

### Eligibility Criteria

The inclusion criteria are as follows: (1) age 18-75 years; (2) histologically or cytologically confirmed NSCLC; (3) liver metastases confirmed by abdominal imaging, such as computerized tomography (CT) or magnetic resonance imaging (MRI); (4) negative for epidermal growth factor receptor and anaplastic lymphoma kinase, as assessed by polymerase chain reaction or next-generation sequencing; (5) Eastern Cooperative Oncology Group performance status 0-2; (6) at least 2 measurable lesions according to Response Evaluation Criteria in Solid Tumors 1.1 (RECIST1.1) guidelines ; (7) asymptomatic (identified as being without significant symptoms at baseline, judged by the investigator as not requiring steroids and/or local treatment) or symptomatically stable brain metastases (without significant symptoms after local treatment, such as RT, and at least 7 days free from steroids and/or anticonvulsant therapy at the time of enrollment); (8) requirement for immunotherapy; (9) liver metastases (confirmed by imaging and multidisciplinary tumor board consultation) that can be treated with HIFU; (10) agreement to use effective contraception during the beginning of the trial and 90 days after the dose; (11) written informed consent.

Exclusion criteria for patients are as follows: (1) mixed lung cancer, including neuroendocrine tumors or sarcoma; (2) history of other malignancies within 5 years; (3) effusions of the third space that cannot be drained or otherwise treated; 4) severe interstitial pneumonia or severe diffusion dysfunction; (4) liver function with Child-Pugh grade C; (5) history of immunodeficiency disease necessitating long-term treatment with glucocorticoids; (6) history of acute cardiovascular or cerebrovascular disorders, including acute cerebral infarction and acute coronary syndrome, within the last month; (7) pregnant or lactating women; (8) life expectancy 3 months or less.

### Intervention

**Figure 2:**
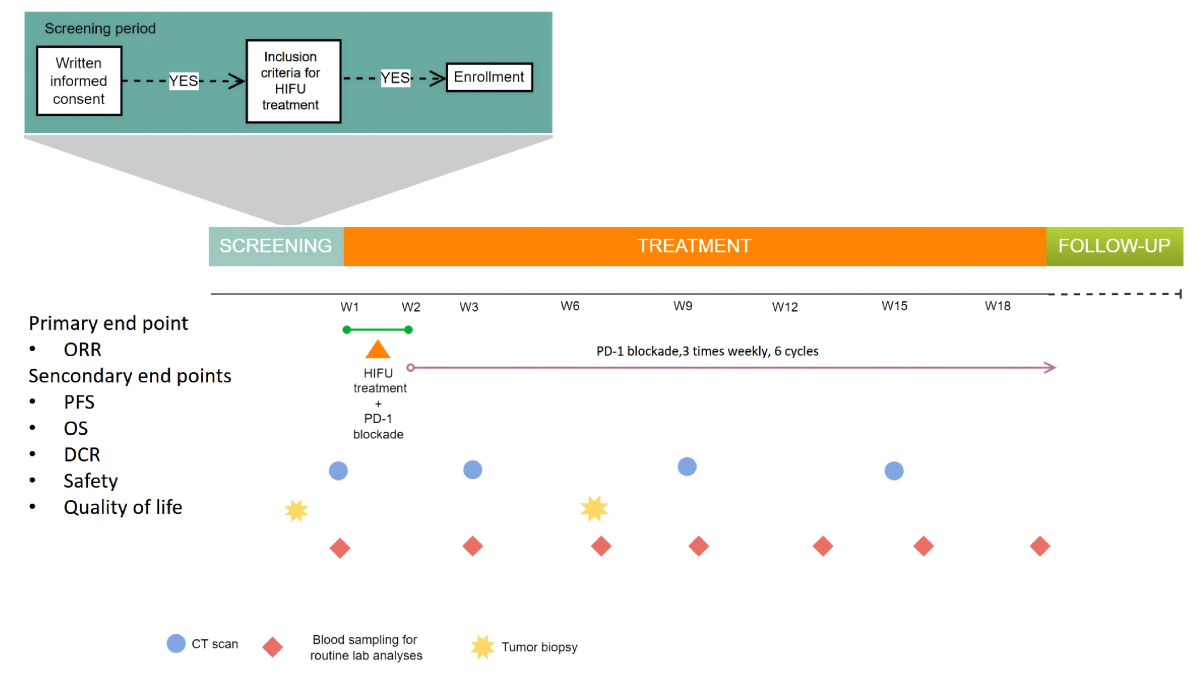
Details of the scheduling for all planned blood sampling and tumor imaging operations during the trial to document the treatment’s safety and efficacy. Palliative radiation therapy for liver metastases and other immunotherapy treatments will not be performed. CT: computerized tomography; DCR: disease control rate; HIFU: high-intensity focused ultrasound; ORR: overall response rate; PD: programmed cell death protein; PFS: progression free survival.

Eligible patients will receive HIFU on liver metastases and PD-1 blockade therapy. Given the potential immunomodulation effect of HIFU, ablation of metastatic liver lesions was planned to take place within no more than 7 days of immunotherapy. The PD-1 blockade was injected repeatedly every 3 weeks for 6 cycles. The combination of immunotherapy and ablation was administered once in week 1. The study schema provides a comprehensive summary (Figure 2). Patients who are considered to have obtained clinical benefits, including a complete response (CR), partial response (PR), or having achieved stable disease (SD), will be allowed to continue PD-1–blocking medicines (every 3 weeks) for up to 2 years beyond the end of the trial. Patients with progressive disease will exit the trial or undergo re-ablation with HIFU according to their choice. Re-ablation will be performed in liver metastases without changing the immunotherapy regimen, and the efficacy will be evaluated 3 weeks later. If the treatment outcome is progressive disease, the patient will be withdrawn from the study. If the outcome is SD, PR, or CR, the patient will be treated and followed up. The standard PD-1 blockade treatment plan for advanced lung cancer with liver metastases will be used, as determined by the investigators and based on clinical guidelines.

Whole blood, plasma, archived cancer tissue, and tumor biopsies will be taken for exploratory research. Given the stability and availability of the test, peripheral blood will be obtained for lymphocyte subsets and the levels of 12 cytokines (tumor necrosis factor [TNF]-α, interleukin [IL]-6, IL-10, IL-17, interferon [IFN]-α, IL-1β, IL-2, IL-5, IL-8, IL-12P70, IFN-γ, IL-4) will be measured before and after HIFU treatment. Tumor biopsy tissue will be embedded in paraffin and analyzed for immune cell infiltration and PD-L1 expression. This information will help us determine whether the medication modifies the diversity of immune response and whether these changes are reflected in the blood. This could aid in the selection of predictive biomarkers for patients in future trials by connecting immunological alterations to clinical symptoms.

### Focused Ultrasound Ablation

Patients were chosen according to the following HIFU treatment inclusion criteria: (1) suitability for anesthesia and sedation, (2) visibility in diagnostic sonography, (3) accessibility (maximum of 11 cm between the skin and the deepest tumor sections), (4) absence of extensive scarring or extensive calcifications along the acoustic route, and (5) absence of metal clips following surgery. Ultrasound-guided HIFU will be performed with a HIFU system (the Focused Ultrasound Tumor Therapeutic System [model-JC200]; Chongqing Haifu Medical Technology Co Ltd), with the patient in a lateral position. Operational details were chosen with reference to previous studies [25,26]. Prior to HIFU treatment, ultrasound localization is required to assess the safety of the patient’s acoustic channel and the accessibility of the treatment target area to determine the treatment regimen. Gastrointestinal preparation is required the day before HIFU treatment, similar to the preparation before colonoscopy. To minimize skin damage caused by HIFU, the treatment area should be shaved and moisturized on the day of surgery. The patient is given general anesthesia by a qualified anesthesiologist. During treatment, ultrasound can be used to detect the distance between the treated lesion and the edge of the lesion and the surrounding vulnerable structures (eg, the intestinal and biliary stents) in real time to prevent complications caused by heat. The power of HIFU ablation will be adjusted in real time according to the treatment.

If a patient has multiple liver metastases, HIFU ablation should not be performed on more than 3 lesions at once to ensure patient safety and tolerance. If there are more than 3 liver metastases, it is possible to ablate the largest 3 first, monitor the remaining lesions, and ablate them again if necessary.

### End Points

Overall response rate (ORR) is the primary end point. The secondary end points are PFS, OS, disease control rate, safety, and patient-reported outcomes, including the results of the European Organization for Research and Treatment of Cancer Quality of Life Questionnaire–Core 30 and Quality of Life Questionnaire–Lung Cancer Module 13. MRI or CT examinations will be administered at the start point, week 3, and each 6 weeks following. According to the RECIST1.1 and the National Cancer Institute Common Terminology Criteria for Adverse Events (version 5.0), tumor response and adverse events will be evaluated and recorded during therapy and within 28 days after the last dose, respectively. Telephone checks will be conducted every 3 months until the end of the trial, death, or loss to follow-up.

After illness progression or withdrawal from the trial’s treatment, the use of anticancer medication will be recorded. ORR is the proportion of enrolled patients with a CR or PR based on imaging. PFS is the length of time between enrollment and the occurrence of disease progression. OS is the duration between enrollment and death.

### Monitoring

The effectiveness and safety of the treatment will be evaluated by an independent reviewer annually. The monitoring committee will monitor the trial’s safety, adherence to protocol, and progress and will modify the protocol as necessary and determine the need for early termination of treatment.

### Statistical Analysis

The 2-stage minimax design of Simon was chosen for sample size estimation. With an ORR of 17% or less, HIFU with PD-1 blocking as a combination therapy will be deemed useless or uninteresting; With an ORR of at least 37%, this regimen will warrant further investigation. At a significance level of .05, an analysis with 80% power will require a sample size of at least 30 patients, with 14 during the initial phase and 16 in the next. At the end of the first stage, if 2 or more patients have responded, the second stage can be carried out. If this does not happen, the study has to end. At the end of the study, if at least 8 of 30 patients respond, it will be taken to indicate that the combination therapy is beneficial and worthy of further investigation.

In accordance with the concept of “intention to treat,” all individuals who are given a minimum of 1 injection of the trial medication and undergo effectiveness assessment will be included in efficacy analyses. All patients who are injected with a minimum of 1 dose of the research medication and have a minimum of 1 safety report will be included in the safety analysis. Once we have recorded baseline features, tumor responses, patient-reported outcomes, and adverse events, we will produce descriptive statistics, including Kaplan-Meier survival curves. There will be no data imputation for missing data.

## Results

The study was funded on March 14, 2022, and received ethical approval on April 27, 2022. Clinical trial registration was completed by June 10, 2022, with participant recruitment beginning on July 10, 2022. Data collection commenced on July 14, 2022, with the enrollment of the first patient. By April 2024, 6 participants had been recruited. The results are expected to be published in December 2026.

## Discussion

The HILL study is a phase 2 single-arm trial investigating the efficacy and safety of combining HIFU with PD-1 blockade immunotherapy in patients with lung cancer and liver metastases. Although ICB has yielded promising response rates in lung cancer, its impact on liver metastases remains constrained by the unique immune microenvironment of the liver. This challenge has spurred the search for more effective therapeutic strategies [27]. Local treatments such as RT can modify the liver’s tumor microenvironment, potentially enhancing the effects of ICB-based therapies. As a minimally invasive, repeatable approach, HIFU has seen extensive use in the treatment of solid tumors. We hypothesize that the combination of HIFU with PD-1 inhibition may enhance the clinical response in patients with lung cancer liver metastases. Indeed, HIFU has demonstrated the ability to modulate the tumor microenvironment and stimulate antitumor immunity in both preclinical and clinical settings [28-30].

Preclinical studies across various tumor types demonstrate that HIFU, when combined with immunotherapy, increases the expression of IFN-γ and granzyme B in the distant tumor microenvironment, enhances CD8+ T cell activation, augments tumor cell lysis, and extends survival [24,31-33]. These findings suggest that HIFU may potentiate the effects of immunotherapy. A study by Yang et al [34] involving 14 patients with liver metastases from malignancies treated with HIFU and immunotherapy found that this combination was feasible and safe, achieving an ORR of 21.4%. However, only 3 participants had lung cancer, limiting conclusions about the combination’s effectiveness specifically for lung cancer liver metastases.

Peripheral blood samples are frequently used in clinical trials for biomarker identification due to the challenge of obtaining tissue specimens. To ensure reliability and accessibility in clinical settings, we selected 12 cytokines and lymphocyte subpopulations as biomarkers. These markers are part of routine clinical testing and ensure consistency in results. Clinically relevant findings from these analyses could be quickly implemented. Lymphocyte subpopulations—including T cells, B cells, and natural killer cells—are measured to reflect immune changes, as peripheral blood lymphocytes correlate with immunotherapy efficacy and may reflect synergistic effects of combined treatments [35,36]. Cytokines play a critical role in cancer-immune interactions, influencing multiple aspects of metastasis [37]. The 12 cytokines studied are linked to T cells and macrophages—key immune cells within the tumor microenvironment—and provide an indirect assessment of antitumor treatment efficacy. Therefore, investigating changes in these cytokines during therapy may yield clinically valuable biomarkers.

This study uses HIFU, a local treatment widely used for liver cancer that causes minimal tissue damage and uses external ultrasonic focusing and real-time monitoring, ensuring a high degree of safety and allowing for repeated applications. HIFU’s influence on the tumor microenvironment can activate antitumor immunity, potentially enhancing the benefits of immunotherapy for patients with lung cancer liver metastases. The trial aims to assess the safety and efficacy of this combination therapy, offering a potential new treatment approach. Additionally, biomarker analysis will seek to identify markers that could improve future therapeutic outcomes. However, the study has limitations. The small sample size may affect the generalizability of the results. The single-arm design could introduce bias, and while HIFU and PD-1 blockade have shown promise individually, their combined efficacy and safety remain to be fully determined.

In summary, this study targets an unmet clinical need for patients with lung cancer liver metastases, taking into account their distinct pathophysiology and the limited benefits of immunotherapy in this population. By investigating the effects of HIFU combined with immunotherapy on the liver tumor microenvironment, this trial seeks to provide novel therapeutic options for these patients

## Abbreviations

CD: cluster of differentiation
CR: complete response
CT: computerized tomography
HIFU: high-intensity focused ultrasound
HILL: Ultrasound-Guided High-Intensity Focused Ultrasound Combined With PD-1 Blockade in Patients With Liver Metastases From Lung Cancer
ICB: immune checkpoint blockade
IFN: interferon
IL: interleukin
MRI: magnetic resonance imaging
NSCLC: non–small cell lung cancer
ORR: overall response rate
OS: overall survival
PD: programmed cell death protein
PFS: progression free survival
PR: partial response
RECIST1.1: Response Evaluation Criteria in Solid Tumors 1. 1
RT: radiation therapy
TNF: tumor necrosis factor

## Data Availability

The data that support the findings of this study are available from the corresponding author upon reasonable request.

## References

[1] Thai AA, Solomon BJ, Sequist LV, Gainor JF, Heist RS (2021). Lung cancer. Lancet.

[2] Sung H, Ferlay J, Siegel RL, Laversanne M, Soerjomataram I, Jemal A, Bray F (2021). Global Cancer Statistics 2020: GLOBOCAN estimates of incidence and mortality worldwide for 36 cancers in 185 countries. CA Cancer J Clin.

[3] Ragavan M, Das M (2020). Systemic therapy of extensive stage small cell lung cancer in the era of immunotherapy. Curr Treat Options Oncol.

[4] Yang S, Zhang Z, Wang Q (2019). Emerging therapies for small cell lung cancer. J Hematol Oncol.

[5] Riihimäki M, Hemminki A, Fallah M, Thomsen H, Sundquist K, Sundquist J, Hemminki K (2014). Metastatic sites and survival in lung cancer. Lung Cancer.

[6] Vokes EE, Ready N, Felip E, Horn L, Burgio MA, Antonia SJ, Arén Frontera O, Gettinger S, Holgado E, Spigel D, Waterhouse D, Domine M, Garassino M, Chow LQM, Blumenschein G, Barlesi F, Coudert B, Gainor J, Arrieta O, Brahmer J, Butts C, Steins M, Geese WJ, Li A, Healey D, Crinò L (2018). Nivolumab versus docetaxel in previously treated advanced non-small-cell lung cancer (CheckMate 017 and CheckMate 057): 3-year update and outcomes in patients with liver metastases. Ann Oncol.

[7] Tumeh P, Hellmann Matthew D, Hamid Omid, Tsai Katy K, Loo Kimberly L, Gubens Matthew A, Rosenblum Michael, Harview Christina L, Taube Janis M, Handley Nathan, Khurana Neharika, Nosrati Adi, Krummel Matthew F, Tucker Andrew, Sosa Eduardo V, Sanchez Phillip J, Banayan Nooriel, Osorio Juan C, Nguyen-Kim Dan L, Chang Jeremy, Shintaku I Peter, Boasberg Peter D, Taylor Emma J, Munster Pamela N, Algazi Alain P, Chmielowski Bartosz, Dummer Reinhard, Grogan Tristan R, Elashoff David, Hwang Jimmy, Goldinger Simone M, Garon Edward B, Pierce Robert H, Daud Adil (2017). Liver metastasis and treatment outcome with anti-PD-1 monoclonal antibody in patients with melanoma and NSCLC. Cancer Immunol Res.

[8] Yu J, Green MD, Li S, Sun Y, Journey SN, Choi JE, Rizvi SM, Qin A, Waninger JJ, Lang X, Chopra Z, El Naqa I, Zhou J, Bian Y, Jiang L, Tezel A, Skvarce J, Achar RK, Sitto M, Rosen BS, Su F, Narayanan SP, Cao X, Wei S, Szeliga W, Vatan L, Mayo C, Morgan MA, Schonewolf CA, Cuneo K, Kryczek I, Ma VT, Lao CD, Lawrence TS, Ramnath N, Wen F, Chinnaiyan AM, Cieslik M, Alva A, Zou W (2021). Liver metastasis restrains immunotherapy efficacy via macrophage-mediated T cell elimination. Nat Med.

[9] Antonia SJ, Borghaei H, Ramalingam SS, Horn L, De Castro Carpeño Javier, Pluzanski A, Burgio MA, Garassino M, Chow LQM, Gettinger S, Crinò Lucio, Planchard D, Butts C, Drilon A, Wojcik-Tomaszewska J, Otterson GA, Agrawal S, Li A, Penrod JR, Brahmer J (2019). Four-year survival with nivolumab in patients with previously treated advanced non-small-cell lung cancer: a pooled analysis. Lancet Oncol.

[10] Ciner AT, Jones K, Muschel RJ, Brodt P (2021). The unique immune microenvironment of liver metastases: challenges and opportunities. Semin Cancer Biol.

[11] Doherty D (2016). Immunity, tolerance and autoimmunity in the liver: a comprehensive review. J Autoimmun.

[12] Oura K, Morishita A, Tani J, Masaki T (2021). Tumor immune microenvironment and immunosuppressive therapy in hepatocellular carcinoma: a review. Int J Mol Sci.

[13] Yu J, Green MD, Li S, Sun Y, Journey SN, Choi JE, Rizvi SM, Qin A, Waninger JJ, Lang X, Chopra Z, El Naqa I, Zhou J, Bian Y, Jiang L, Tezel A, Skvarce J, Achar RK, Sitto M, Rosen BS, Su F, Narayanan SP, Cao X, Wei S, Szeliga W, Vatan L, Mayo C, Morgan MA, Schonewolf CA, Cuneo K, Kryczek I, Ma VT, Lao CD, Lawrence TS, Ramnath N, Wen F, Chinnaiyan AM, Cieslik M, Alva A, Zou W (2021). Liver metastasis restrains immunotherapy efficacy via macrophage-mediated T cell elimination. Nat Med.

[14] Kubes P, Jenne C (2018). Immune Responses in the Liver. Annu Rev Immunol.

[15] Chicas-Sett R, Morales-Orue I, Castilla-Martinez J, Zafra-Martin J, Kannemann A, Blanco J, Lloret M, Lara PC (2019). Stereotactic ablative radiotherapy combined with immune checkpoint inhibitors reboots the immune response assisted by immunotherapy in metastatic lung cancer: a systematic review. Int J Mol Sci.

[16] Golden E, Demaria Sandra, Schiff Peter B, Chachoua Abraham, Formenti Silvia C (2013). An abscopal response to radiation and ipilimumab in a patient with metastatic non-small cell lung cancer. Cancer Immunol Res.

[17] Pagès Franck, Kirilovsky A, Mlecnik B, Asslaber M, Tosolini M, Bindea G, Lagorce C, Wind P, Marliot F, Bruneval P, Zatloukal K, Trajanoski Z, Berger A, Fridman W, Galon J (2009). In situ cytotoxic and memory T cells predict outcome in patients with early-stage colorectal cancer. J Clin Oncol.

[18] Galon J, Bruni D (2019). Approaches to treat immune hot, altered and cold tumours with combination immunotherapies. Nat Rev Drug Discov.

[19] Rix A, Lederle Wiltrud, Theek Benjamin, Lammers Twan, Moonen Chrit, Schmitz Georg, Kiessling Fabian (2018). Advanced ultrasound technologies for diagnosis and therapy. J Nucl Med.

[20] Izadifar Z, Izadifar Z, Chapman D, Babyn P (2020). An introduction to high intensity focused ultrasound: systematic review on principles, devices, and clinical applications. J Clin Med.

[21] Panzone J, Byler T, Bratslavsky G, Goldberg H (2022). Applications of focused ultrasound in the treatment of genitourinary cancers. Cancers (Basel).

[22] Tsukamoto S, Kido A, Tanaka Y, Facchini G, Peta G, Rossi G, Mavrogenis AF (2021). Current overview of treatment for metastatic bone disease. Curr Oncol.

[23] Eranki Avinash, Srinivasan Priya, Ries Mario, Kim AeRang, Lazarski Christopher A, Rossi Christopher T, Khokhlova Tatiana D, Wilson Emmanuel, Knoblach Susan M, Sharma Karun V, Wood Bradford J, Moonen Chrit, Sandler Anthony D, Kim Peter C W (2020). High-intensity focused ultrasound (HIFU) triggers immune sensitization of refractory murine neuroblastoma to checkpoint inhibitor therapy. Clin Cancer Res.

[24] Silvestrini M, Ingham Elizabeth S, Mahakian Lisa M, Kheirolomoom Azadeh, Liu Yu, Fite Brett Z, Tam Sarah M, Tucci Samantha T, Watson Katherine D, Wong Andrew W, Monjazeb Arta M, Hubbard Neil E, Murphy William J, Borowsky Alexander D, Ferrara Katherine W (2017). Priming is key to effective incorporation of image-guided thermal ablation into immunotherapy protocols. JCI Insight.

[25] Ng K, Poon Ronnie T P, Chan See Ching, Chok Kenneth S H, Cheung Tan To, Tung Helen, Chu Ferdinand, Tso Wai Kuen, Yu Wan Ching, Lo Chung Mau, Fan Sheung Tat (2011). High-intensity focused ultrasound for hepatocellular carcinoma: a single-center experience. Ann Surg.

[26] Chok KSH, Cheung TT, Lo RCL, Chu FSK, Tsang SHY, Chan ACY, Sharr WW, Fung JYY, Dai WC, Chan SC, Fan ST, Lo CM (2014). Pilot study of high-intensity focused ultrasound ablation as a bridging therapy for hepatocellular carcinoma patients wait-listed for liver transplantation. Liver Transpl.

[27] Reck M, Mok TSK, Nishio M, Jotte RM, Cappuzzo F, Orlandi F, Stroyakovskiy D, Nogami N, Rodríguez-Abreu Delvys, Moro-Sibilot D, Thomas CA, Barlesi F, Finley G, Lee A, Coleman S, Deng Y, Kowanetz M, Shankar G, Lin W, Socinski MA, IMpower150 Study Group (2019). Atezolizumab plus bevacizumab and chemotherapy in non-small-cell lung cancer (IMpower150): key subgroup analyses of patients with EGFR mutations or baseline liver metastases in a randomised, open-label phase 3 trial. Lancet Respir Med.

[28] Joiner Jordan B, Pylayeva-Gupta Yuliya, Dayton Paul A (2020). Focused ultrasound for immunomodulation of the tumor microenvironment. J Immunol.

[29] Cirincione R, Di Maggio FM, Forte GI, Minafra L, Bravatà Valentina, Castiglia L, Cavalieri V, Borasi G, Russo G, Lio D, Messa C, Gilardi MC, Cammarata FP (2017). High-intensity focused ultrasound- and radiation therapy-induced immuno-modulation: comparison and potential opportunities. Ultrasound Med Biol.

[30] Shi G, Zhong M, Ye F, Zhang X (2019). Low-frequency HIFU induced cancer immunotherapy: tempting challenges and potential opportunities. Cancer Biol Med.

[31] Li Ting-Chuan, Liu Chih-Chun, Lee Yan-Zhang, Hsu Yu-Hone, Chiang Chi-Feng, Miaw Shi-Chuen, Lin Win-Li (2020). Combination therapy of pulsed-wave ultrasound hyperthermia and immunostimulant OK-432 enhances systemic antitumor immunity for cancer treatment. Int J Radiat Oncol Biol Phys.

[32] Tang J, Tang J, Li H, Zhou J, Tang N, Zhu Q, Wang X, Zhu B, Li N, Liu Z (2023). Mechanical destruction using a minimally invasive ultrasound needle induces anti-tumor immune responses and synergizes with the anti-PD-L1 blockade. Cancer Lett.

[33] Qu S, Worlikar T, Felsted AE, Ganguly A, Beems MV, Hubbard R, Pepple AL, Kevelin AA, Garavaglia H, Dib J, Toma M, Huang H, Tsung A, Xu Z, Cho CS (2020). Non-thermal histotripsy tumor ablation promotes abscopal immune responses that enhance cancer immunotherapy. J Immunother Cancer.

[34] Yang X, Liao Y, Fan L, Lin B, Li J, Wu D, Liao D, Yuan L, Liu J, Gao F, Feng G, Du X (2024). High-intensity focused ultrasound ablation combined with immunotherapy for treating liver metastases: a prospective non-randomized trial. PLoS One.

[35] Fujikawa K, Saito T, Kurose K, Kojima T, Funakoshi T, Sato E, Kakimi K, Iida S, Doki Y, Oka M, Ueda R, Wada H (2023). Integrated analysis of phase 1a and 1b randomized controlled trials; Treg-targeted cancer immunotherapy with the humanized anti-CCR4 antibody, KW-0761, for advanced solid tumors. PLoS One.

[36] Chun B, Pucilowska J, Chang S, Kim I, Nikitin B, Koguchi Y, Redmond WL, Bernard B, Rajamanickam V, Polaske N, Fields PA, Conrad V, Schmidt M, Urba WJ, Conlin AK, McArthur HL, Page DB (2022). Changes in T-cell subsets and clonal repertoire during chemoimmunotherapy with pembrolizumab and paclitaxel or capecitabine for metastatic triple-negative breast cancer. J Immunother Cancer.

[37] Lan T, Chen L, Wei X (2021). Inflammatory cytokines in cancer: comprehensive understanding and clinical progress in gene therapy. Cells.

